# A Humanized Mouse Model of Tuberculosis

**DOI:** 10.1371/journal.pone.0063331

**Published:** 2013-05-17

**Authors:** Veronica E. Calderon, Gustavo Valbuena, Yenny Goez, Barbara M. Judy, Matthew B. Huante, Putri Sutjita, R. Katie Johnston, D. Mark Estes, Robert L. Hunter, Jeffrey K. Actor, Jeffrey D. Cirillo, Janice J. Endsley

**Affiliations:** 1 Department of Pathology, University of Texas Medical Branch (UTMB), Galveston, Texas, United States of America; 2 Department of Microbiology and Immunology, University of Texas Medical Branch (UTMB), Galveston, Texas, United States of America; 3 University of Georgia, Athens, Georgia, United States of America; 4 University of Texas-Houston Health Science Center, Department of Pathology, Houston, Texas, United States of America; 5 Texas A&M Health Sciences Center, Department of Microbial and Molecular Pathogenesis, College Station, Texas, United States of America; Fundació Institut d’Investigació en Ciències de la Salut Germans Trias i Pujol. Universitat Autònoma de Barcelona. CIBERES, Spain

## Abstract

*Mycobacterium tuberculosis* (*M.tb*) is the second leading infectious cause of death worldwide and the primary cause of death in people living with HIV/AIDS. There are several excellent animal models employed to study tuberculosis (TB), but many have limitations for reproducing human pathology and none are amenable to the direct study of HIV/*M.tb* co-infection. The humanized mouse has been increasingly employed to explore HIV infection and other pathogens where animal models are limiting. Our goal was to develop a small animal model of *M.tb* infection using the bone marrow, liver, thymus (BLT) humanized mouse. NOD-SCID/γ_c_^null^ mice were engrafted with human fetal liver and thymus tissue, and supplemented with CD34^+^ fetal liver cells. Excellent reconstitution, as measured by expression of the human CD45 pan leukocyte marker by peripheral blood populations, was observed at 12 weeks after engraftment. Human T cells (CD3, CD4, CD8), as well as natural killer cells and monocyte/macrophages were all observed within the human leukocyte (CD45^+^) population. Importantly, human T cells were functionally competent as determined by proliferative capacity and effector molecule (e.g. IFN-γ, granulysin, perforin) expression in response to positive stimuli. Animals infected intranasally with *M.tb* had progressive bacterial infection in the lung and dissemination to spleen and liver from 2–8 weeks post infection. Sites of infection in the lung were characterized by the formation of organized granulomatous lesions, caseous necrosis, bronchial obstruction, and crystallization of cholesterol deposits. Human T cells were distributed throughout the lung, liver, and spleen at sites of inflammation and bacterial growth and were organized to the periphery of granulomas. These preliminary results demonstrate the potential to use the humanized mouse as a model of experimental TB.

## Introduction

Tuberculosis, caused by *M.tb*, is a major global health threat. Approximately 2 billion people (one-third of the world’s population) are estimated to be latently infected and nearly 9 million people became newly infected in 2011 [1]. *M.tb* is the second leading infectious cause of death worldwide and the leading cause of death in people with HIV/AIDS [1]. Of great concern is the growing incidence of multi- and extensively drug resistant isolates of *M.tb* associated with case mismanagement and immune compromise due to HIV infection [1]–[3]. There is thus an urgent need to develop and test new vaccines and drug compounds to prevent and treat TB. Towards this end, development of additional animal models to complement existing models and allow new avenues of discovery is needed.

Several excellent animal models are available to study *M.tb* infection, including mice, guinea pigs, rabbits, cattle, and non-human primates (NHP) [4]–[9]. Mice are the most widely used model because they are easy to use, inexpensive, and reagents are readily available. An important limitation of this model, though, is the lack of granuloma formation similar to human infection [6], [9]–[11]. The NHP, rabbit, and guinea pigs develop necrotic lesions similar to human TB disease [12]. Immunological reagents are limiting for the rabbit, guinea pig, and cow, however; and, like the mouse, these models are not amenable to the study of HIV/*M.tb* co-infection.

A broad spectrum of TB disease states can develop in the NHP, and co-infection can be simulated using simian immunodeficiency virus (SIV) and *M.tb* [5], [13], [14]. Infection with SIV reproduces many clinical features of HIV and SIV/*M.tb* co-infection promotes aggressive TB disease and reactivation in latency models [13]–[15]. The significant cost (animals, housing, personnel) as well as regulatory issues limit the use of the NHP co-infection model on broader scale. Further, there are significant differences between SIV and HIV including genetic heterogeneity and receptor usage for host cell entry [16]. Thus, there is a need for a small animal model that is: less expensive, available in larger numbers, does not require specialized facilities and staff, can take advantage of the availability of human reagents, and can be infected with HIV as compared to SIV.

The development of the humanized BLT mice has recently opened new avenues of study for important human diseases. Several studies have demonstrated the potential to utilize this model to study HIV virus [17]–[20] and a few other pathogens where host tropism limits use of other animal models [21]–[23]. Currently, the humanized BLT mouse has not been developed for *M.tb* infection and there is no small animal model to study co-infections, such as HIV/*M.tb*. Such a model is urgently needed to improve our understanding of the human immune response to *M.tb,* advance our knowledge of HIV/*M.tb* co-infection pathobiology, inform vaccine development, and guide drug development to reduce side effects and drug interactions.

Our studies demonstrate that the humanized BLT mouse develops TB and displays pathology similar to that observed in infected humans. We show here that humanized mice were successfully reconstituted with human leukocytes (including T cells, macrophages, natural killer cells, and antigen presenting cells). Following *M.tb* infection, humanized BLT mice displayed progressive and disseminated bacterial infection, and a spectrum of organized lesions with necrotic centers at sites of infection along with cholesterol crystal deposits and bronchial obstruction. We further demonstrate that human T cells (CD4^+^ as well as CD8^+^) and monocytes/macrophages are distributed normally in tissue. Importantly, human T cells in our mouse model proliferate in response to activation, express effector molecules (e.g. IFN-γ, granulysin), and are recruited to and organized at sites of infection. These preliminary studies support the exciting possibility of using this small animal model to understand the human immune response to *M.tb* and indicate the potential to develop a HIV/*M.tb* co-infection model.

## Results

### Production of the Humanized BLT Mouse Model to Study TB

There are several different mouse models that are generally described as “humanized” based on transgenic expression of human genes, adoptive transfer of human PBLs, human stem cell transplant, and many more. For a full description and comparison of these models please see the following reviews [24], [25]. Of these models, the bone marrow, liver, thymus (or BLT) described by J.V. Garcia and colleagues [26], and reproduced here, is the most difficult to generate because of the requirement for surgical engraftment of human fetal tissue in the mouse kidney capsule.

We routinely engraft 35 to 40 mice with human fetal tissues from an individual donor for various studies by members of our collaborative group. The average reconstitution level varies from donor to donor but is in the 30 to 50% range across many groups. Individuals within each cohort can have relatively large variations in reconstitution levels with ranges between 5 to 80% not being unusual; similar findings have been reported by others. Animals are individually identified so that differences in experimental outcome can be assessed relative to reconstitution. For many experimental outcomes, the differences in the levels of reconstitution do not have a major impact. For most experiments, we use humanized mice produced from different donors to compensate for individual variations. Of course, numbers of animals needed to obtain robust data depend on a power analysis and expected meaningful differences between groups. Depending on the type of experiment, animals can be blocked by donor and/or ranges in the level of reconstitution to decrease the sources of variation in the statistical analysis.

**Figure 1:**
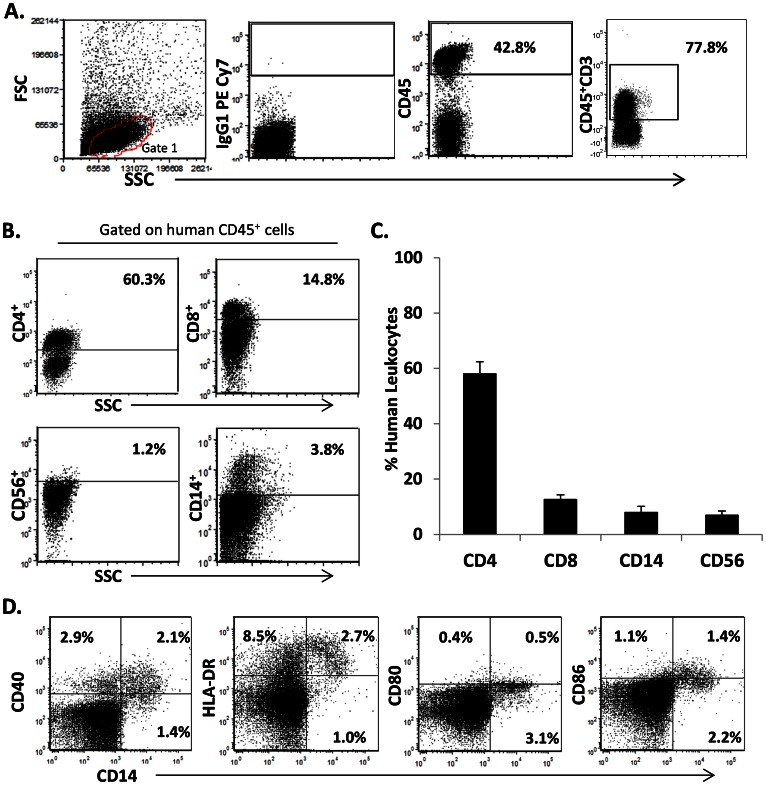
Production of the humanized BLT mouse to study TB. NOD/SCID/γ_c_^null^ (NSG) mice were engrafted with human fetal liver and thymus, and supplemented with CD34^+^ cells. Shown in A, flow cytometry analysis displaying side scatter (SSC) and forward scatter (FSC) characteristics (Gate 1) of isolated peripheral blood from a representative BLT mouse twelve weeks post-engraftment. Plots 2–4 are the gating strategy for selection of cells expressing human CD45 pan leukocyte marker, the corresponding isotype control (IgG1 PE Cy7), and the CD3^+^ population subgate. B, percentage of the gated cells expressing markers for T cell subsets (CD4, CD8), NK cells (CD3^−^CD56^+^) and monocyte/macrophages (CD14^+^). C, average leukocyte % among gated CD45 cells in four groups of reconstituted BLT mice (n = 44) used for the subsequent studies (Fig. 2–8). D shows the expression of antigen presenting cells (APC) markers relevant to antigen presentation (HLA-DR) and T cell activation (CD40, CD80, CD86) expressed by peripheral blood monocyte/macrophages.

**Figure 2:**
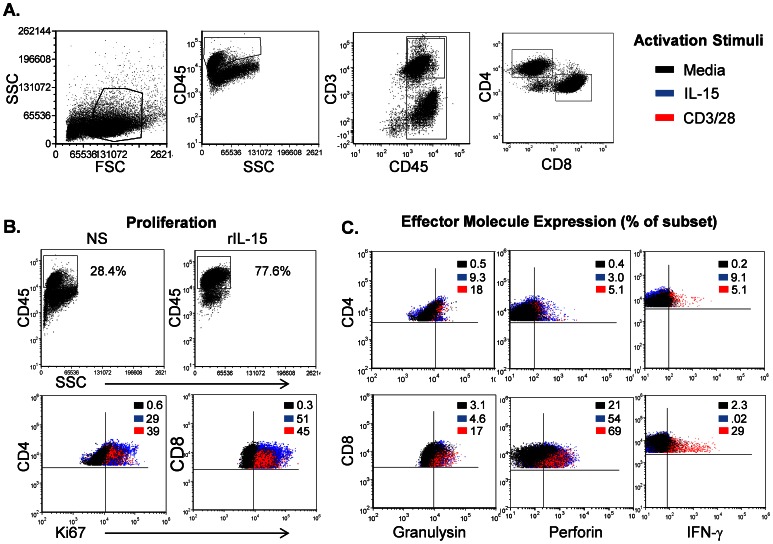
Functional potential of splenic T cell populations in the BLT mouse. Spleens were disrupted to single-cell suspensions and activated with control (media), antibodies to human CD3/CD28, or rIL-15 (15 ng/ml) for 5 days. Following activation, the surface expression of cellular phenotype markers (CD45, CD3, CD4, and CD8) and intracellular proteins (Ki67, granulysin, perforin, and IFN-γ) was detected using flow cytometry. Shown in A are side scatter and forward scatter characteristics of isolated splenocytes (Gate 1), gating strategy to enable analysis of human CD45^+^ and CD3^+^ cell populations and individual T cell (CD4 and CD8) subset gating. B, increase in % of human CD45^+^ cells within the isolated splenocytes following 5 d activation with recombinant IL-15 (15 ng/ml) (upper plots) and activation-induced increase in expression of the proliferation marker Ki67 (lower plots) by treatment displayed as a color dot plot overlay. C, expression of effector molecules (granulysin, perforin, IFN-γ) shown by dot plot overlays demonstrating inducible expression upon activation with CD3/CD28 (red) and rIL-15 (blue) compared to non-activated cells (black). Data shown in A, B, and C is from a representative mouse (n = 3).

Though technically challenging, this model has been reported to achieve reconstitution of both helper and cytotoxic (CTL) T lymphocytes and antigen presenting cells [20], [26], the interactions of which are critical to studies of TB. As shown in Figure 1, 2, and S1, we could successfully reconstitute human immune populations using NOD/SCID/γ_c_^null^ (NSG) mice engrafted with human fetal liver and thymus tissues and CD34^+^ cells as previously described [26]. Twelve weeks post-engraftment, our BLT mice had high levels of human leukocytes (CD45^+^ cells) in peripheral blood (average 27% of the total CD45^+^ cells, n = 44) (Figure 1A). Immediately prior to infection, human leukocytes were further evaluated in the peripheral blood of BLT mice to determine levels of human T cells, T cell subsets (CD4, CD8), and antigen presenting cells (APC). Figure 1A and B demonstrate that a large proportion of the total human leukocytes were T cells (average of 71% of the total human CD45^+^ population), including both CD4 and CD8 T cells subsets. The circulating human leukocyte pool included small numbers of CD3-CD56^+^ Natural Killer (NK) cells (Figure 1B). Human cells expressing the CD14 marker identifying monocyte/macrophages were also observed, in agreement with a previous report [26]. To evaluate the potential for these cells to activate T cells, we further evaluated expression of key surface markers by CD14^+^ cells in this model. As shown in Figure 1D, the CD14^+^ population expresses receptors of importance for antigen presentation (HLA-DR) and lymphocyte activation (CD40, CD80, and CD86).

### Tissue Distribution of Human Leukocytes in Humanized BLT Mice

TB is primarily a disease of the lung; however, the bacteria can additionally disseminate to and cause immune-mediated pathology in many organs. To assess the potential for human leukocytes to migrate to tissues, we evaluated leukocyte populations in the lung, spleen, and liver tissues of BLT humanized mice (Figure S1). Organs were harvested following reconstitution (≥12 wk), processed to single-cell suspensions, and labeled with human leukocyte surface phenotype markers (CD45, CD3, CD4, CD8, CD14, CD56, CD1a), or isotype-matched nonspecific antibody as a control, and analyzed by flow cytometry. Human leukocytes (CD45^+^ cells) were abundant in the spleen (Figure S1) of non-infected BLT mice and could also be found in the lung and liver. T cells (CD3^+^) were especially noted in the spleen and liver, while a large population of cells with a monocyte/macrophage phenotype (CD14) was noted in the lung. Cells with a tissue DC phenotype (CD45^+^CD14^−^CD1a^+^) were also observed in the lung, liver, and spleen (data not shown) similar to reports by Melkus, et al, [26].

Both CD4^+^ and CD8^+^ T cell subsets were represented in all 3 tissues, while NK cells (CD56^+^), though low, were most abundant in the spleen, similar to previous reports with CD34^+^-reconstituted NSG mice (Figure S1). In samples from the blood and spleen, the CD4^+^ and CD8^+^ T cell subsets generally accounted for >95% of the total CD3^+^ population. A preliminary experiment with samples from 1 animal demonstrated that human CD3^+^ cells expressing the gamma delta T cell receptor (γδ-TCR) can also develop in our BLT mouse (data not shown) as previously described for non-BLT humanized mouse models [27]. In both blood and tissue, the NK cells and APC populations generally accounted for ∼50% of the CD3- (non-T cell) CD45^+^ cells. This suggests that other human leukocytes, such as B cells, also comprise a large part of the non-T cell population as well. Overall, the distribution of human leukocyte populations that we observed in our model is fairly consistent with those reported by Melkus, et al., [26] though we noted generally less human leukocytes in the liver in our mice (Figure S1).

### Functional Potential of Splenic T Cell Populations in the BLT Mouse

Though human T cells are generally well reconstituted in various humanized mouse models, in other reports these cells have been shown to have functional defects [28], [29]. The BLT model is the most advanced in this regard, and results from a small number of studies show the potential for T cells from these models to recognize antigen, proliferate, and express some human cytokines and Granzyme B upon activation [20], [26], [27]. Herein, we assessed the functional potential of T cells from humanized mice generated in our laboratory and expanded the characterization of the human CTL repertoire that can be activated (Figure 2). Using anti-CD3/CD28 or recombinant IL-15 (rIL-15), we observed a robust proliferative response by both CD4^+^ and CD8^+^ T cells, as measured by intracellular levels of the Ki67 marker (Figure 2 B, lower plots). This effect was also very evident from the change in % of total cells in the human CD45^+^ cell gate following 5 d of activation with rIL-15 (Figure 2B, upper panels). Importantly, both T cell populations expressed augmented levels of the effector molecules IFN-γ, perforin, and granulysin in response to activation as shown by the color dot plot overlays in Figure 2C. Similar to human peripheral blood T cells [30], CD8^+^ T cells constitutively express perforin and granulysin, and can also greatly increase expression of these cytotoxic/antibacterial molecules upon activation. We additionally show that splenic T cells from BLT mice are able to proliferate and increase effector protein expression in response to recombinant human IL-15 (Figure 2C), an important molecule for T cell survival and effector activity.

### Development of *M.tb* Infection in the BLT Mouse

**Figure 3:**
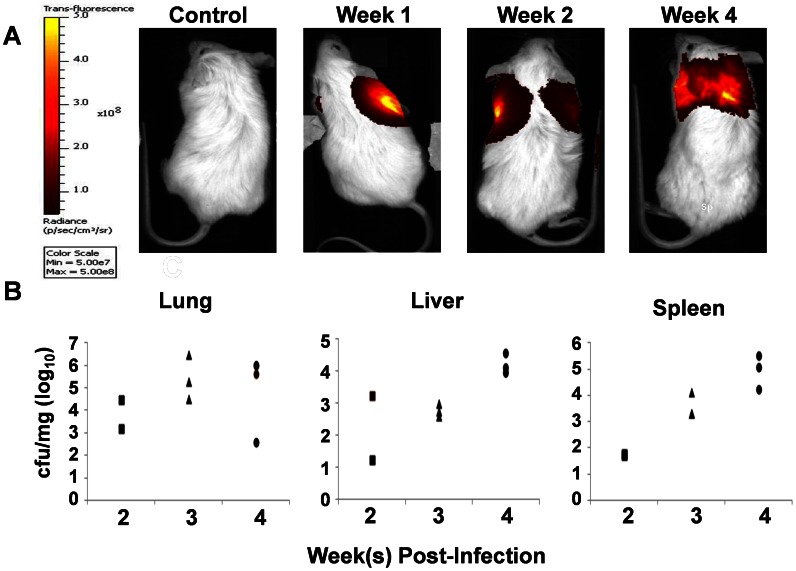
Progression of M.tb infection in humanized BLT mice. Animals were infected i.n. with CFU tdTomato H37RV M.tb and in-vivo imaging (IVIS) performed at weekly time points. Shown in A is the fluorescent intensity signal from a group of animals infected with 106 CFU represented with a pseudocolor scale ranging from yellow (most intense) to dark red (least intense) and IVIS images of BLT mice; across: non-infected control mouse and M.tb infected BLT mice at 1, 2, and 4 weeks p.i. B, bacterial burden (CFU) are shown per milligram (mg) of tissue from individual animals (n = 3 per time point) at 2, 3, and 4 weeks p.i.

Currently, there are no published studies, to our knowledge, describing *M.tb* infection in the BLT, or other humanized mouse models. To evaluate the development of *M.tb*, we began by infecting BLT mice i.n. with a 10^6^ CFU of a strain of *M.tb* that expresses a red fluorescent protein (*tdTomato M.tb* H37Rv) as a proof of concept study. In subsequent experiments, groups of animals (n = 3–5) were infected with logarithmically decreasing doses ranging from 10^6^-10^2^ CFU. Using an *in-vivo* imaging system (IVIS), we were able to monitor progressive bacterial infection as described [31] within the lungs of live mice at weekly intervals (Figure 3A). Compared to the control animals, bacterial infection could occasionally be observed in the lung of *M.tb* infected mice from 1 week onward in groups receiving 10^5^ or 10^6^ CFU (Fig. 3A). In groups infected with lower doses (10^2^,10^3^ or 10^4^ CFU), infection was generally not observable via *in vivo* imaging until 2–3 weeks post-infection (**p.i**.) (data not shown). In all experiments, the sensitivity for *in vivo* imaging declined after 4 weeks, especially at sites of dissemination, presumably due to loss of the plasmid expressing the red fluorescent protein. CFU analysis at weekly time points (Figure 3B) further demonstrates the ability of *M.tb* to progress in the lungs and disseminate to various organs within BLT mice from 2–4 weeks p.i.

### Tissue Pathology in *M.tb*-infected BLT Mice

**Figure 4:**
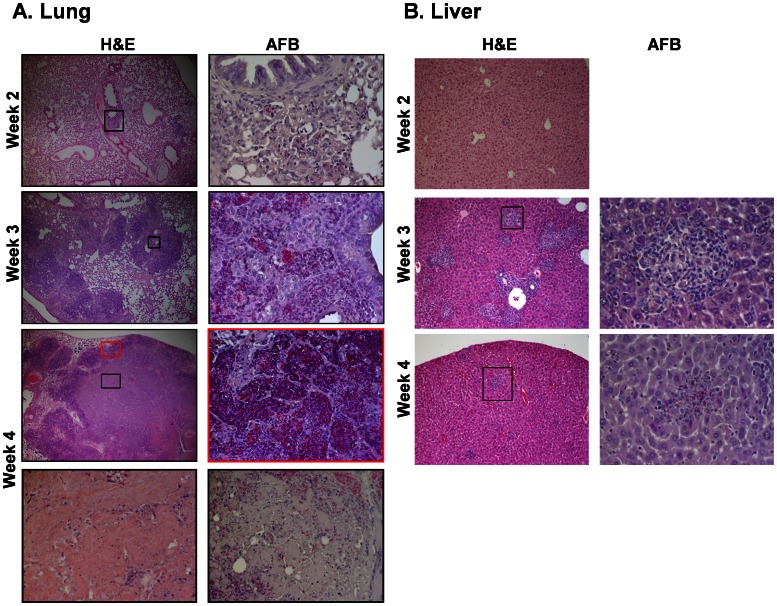
Lung and liver pathology in *M.tb*-infected humanized mice. *M.tb*-infected humanized mice have lung and liver pathology consistent with development of TB. Animals were infected i.n. with 10^6^ CFU *tdTomato* H37RV *M.tb* after verification of appropriate reconstitution with human leukocytes. Shown are images captured by brightfield microscopy following staining of infected tissues using hematoxylin and eosin (**H&E**) and acid-fast stain (Ziehl-Neelson) to detect acid-fast bacilli (**AFB**). The images show tissue damage and inflammation localized to *M.tb* bacilli in formalin-fixed tissue sections of BLT mice infected i.n. with 10^6^ cfu *tdTomato M.tb* H37Rv. Images are representative of mice sacrificed at 2, 3, and 4 wk p.i. described in Fig. 3. Shown in A is the lung tissue pathology visualized by H&E staining (left panels, 4X). Localization and burden of bacilli are shown in right panels (40X) from the region indicated in the H&E image, as visualized by acid fast and hematoxylin staining. B, liver tissue pathology at 2, 3, and 4 weeks p.i. (H&E 10X, AFB 40X of indicated region).

**Figure 5:**
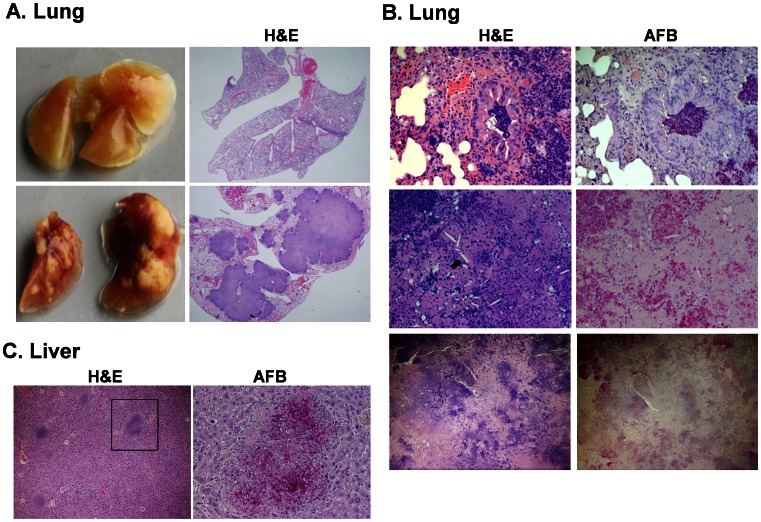
TB in humanized mice infected with a low dose of ***M.tb*** **.** Animals were infected i.n. with 250 CFU *tdTomato* H37RV *M.tb* after verification of appropriate reconstitution with human leukocytes. Shown are images captured by brightfield microscopy following analysis of lung and liver from a representative animal (n = 11). The images demonstrate tissue damage and inflammation (hematoxylin and eosin, H&E) localized to *M.tb* bacilli (acid fast bacilli, AFB) in formalin-fixed tissue sections from lung and liver of BLT mice sacrificed at 6–8 weeks p.i. Shown in A are gross lung lobes (left panels) and cross sections of whole lung stained with H&E, captured using a stereomicroscope. B, Lung tissue pathology visualized by H&E staining (left panels) and AFB (right panels). Top panels (20X) demonstrate bronchial obstruction in the lung and the large numbers of bacteria within the obstruction. Middle panels (20X) show cholesterol crystal deposits (black arrow) observed in large granulomas at later stages (≥6 wk) of infection. Bottom panels (4X) shows center of large, coalescing granulomas characterized by necrosis and lack of AFB. C, shown is liver tissue pathology (left panel, 10X) and AFB (right panel, 40X) in the indicated region). Images are from lung and liver of a representative animal (n = 6).

To evaluate the development of TB in humanized mice, mice were sacrificed at specified time points, and tissue preserved for analysis. The left lung lobe and a lobe of liver, along with the entire spleen, were weighed and used for CFU enumeration. The remaining lung and liver tissue were used to evaluate tissue pathology and bacterial load using light microscopy following hematoxylin and eosin (H&E) and acid fast bacilli (AFB) staining. Figure 4A shows H&E and AFB images at various time points from BLT mice infected with a high dose (10^6^ CFU) of *tdTomato* H37Rv *M.tb*. At 2 weeks p.i., multiple foci of mild interstitial inflammation were observed interspersed with normal areas of lung. The few observed bacteria were contained within areas of inflammation. Bacteria were also observed in the bronchus (data not shown); although inflammatory cell obstruction was not seen at 2 weeks p.i. By week 3 p.i., we observed severe interstitial inflammation along with thickened alveolar walls and perivascular inflammation. Bacterial clustering was evident and primarily contained within these areas of inflammation. Additionally, bronchial obstruction was also observed (data not shown, but shown for a different group in Figure 5). At week 4 p.i., granuloma formation was observed with cellular cuffing and central necrosis. Bacteria were contained in clusters around the periphery of the granuloma within alveolar pockets. Bacterial debris was also seen scattered throughout the necrotic center of the granuloma, along with pycnotic nuclei and cellular fragmentation (Figure 4A). Figure 4B shows liver pathology from BLT humanized mice infected with 10^6^ *tdTomato* H37Rv *M.tb* at various times p.i. At weeks 3 and 4 p.i., multiple areas of organized inflammatory foci are observed throughout the tissue with bacteria relatively contained within the regions of inflammatory response. No significant duct obstruction was noted in the liver, even at 4 weeks p.i.

### *M.tb* Infection in Control NSG Mice without Human Leukocytes Differs from that Observed in Humanized Mice

To evaluate the contribution of the innate immune system of the mouse in our studies, we also infected a small group (n = 4) of non-humanized NSG mice (Figure S2) and used one non-infected NSG mouse as a control. These animals were infected with 10^5^ CFU *tdTomato M.tb* H37Rv instead of 10^6^ due to concerns for a greatly accelerated course of infection due to the lack of an adaptive immune system. Surprisingly, these animals did not all rapidly succumb to disease as expected, given the significant immune deficiencies of these animals. Of the 4 infected animals, 1 expired at <1 week and the remaining animals were euthanized at 2 (n = 1), and 6 (n = 2) weeks. The animal that died at <1 week died as a consequence of anesthesia complications, and was not thought to have succumbed to infection. To assess disease, tissues were harvested at 2 and 6 wk p.i. At week 2 p.i., we observed scattered areas of inflammation in the lung with slight interstitial and alveolar wall thickening (Figure S2A, H&E). Bacterial infection was mostly diffuse with occasional interstitial macrophages containing relatively few bacilli (Figure S2A, AFB). By week 6 p.i., considerable interstitial thickening due to inflammation was observed along with intra-alveolar infection (Figure S2A, H&E). Bacterial clustering was seen and contained to areas of inflammation, primarily within alveolar sacs (Figure S2A, AFB). At this time point, bacterial infection was further seen in the bronchial epithelial wall (data not shown). Within the liver at 2 weeks p.i., normal tissue architecture was observed, along with minor inflammation and relatively few bacilli (Figure S2B). Throughout the liver at week 6 p.i., primarily neutrophilic areas of inflammation were seen containing bacteria (Figure S2B). The extensive granulomatous response observed in the humanized NSG mice was not observed in these mice lacking reconstitution with human cells. Subsequent studies with lower doses of *M.tb* in the BLT mice (Figure 5) confirmed that these observations were not due to differences in dose.

### TB in Humanized Mice Infected with a Low Dose of *M.tb*

Though we observed disease pathology consistent with TB development with high dose infection in our proof of concept studies, lower dose infections with a longer disease course are more relevant to human disease. Preliminary studies with i.n. infections of 10^2^–10^6^ CFU indicated than when lower doses (10^2^ and 10^3^ CFU) were used, poor growth and dissemination was observed when studies were terminated from 1–4 wk p.i. To further evaluate the development of disease with lower dose infection we repeated our studies using i.n. infection with 10^2^ CFU (actual delivered dose of 250 CFU) and extended the time frame of observations. Of these animals (n = 6), 2 animals succumbed early in infection (3 weeks) while the remainder (n = 4) were euthanized from 6–8 weeks p.i. Figure 5A shows the gross lung anatomy from a control non-infected BLT mouse (top) and from an *M.tb* infected BLT mouse (bottom). The H&E image from the *M.tb* infected mouse shows large lung lesions consistent with TB throughout the lung compared to the lung from the control mouse. At 7 weeks p.i. (Figure 5B), granuloma formations were observed in the lung of BLT mice with substantial bacterial clustering peripheral to pathology (Figure 5B). There was little additional change in lung pathology from 6 to 8 wk p.i. (data not shown). Of interest, bronchial obstruction containing large numbers of bacteria and cholesterol crystal formation was observed within *M.tb* infected BLT mouse granulomas in the lung (Figure 5B). In the liver, diffuse areas of inflammation were further observed along with bacilli contained within the pathological foci (Figure 5C).

### Human T Cells are Recruited to and Organize at Sites of Inflammation Following *M.tb* Infection

**Figure 6:**
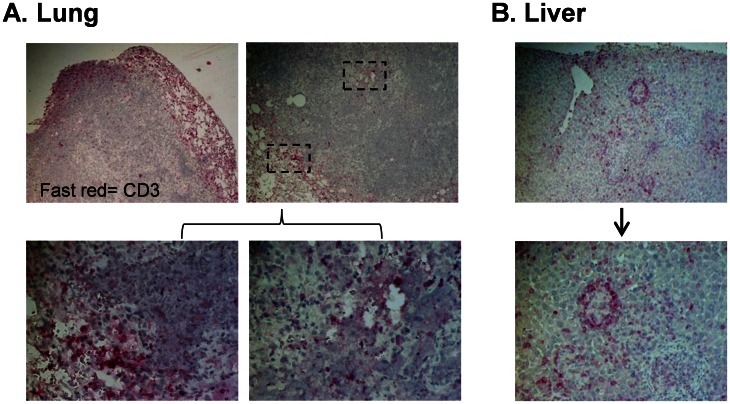
Human T cells are recruited to and organize at lung granulomas and sites of inflammation following *M.tb* infection. Animals were infected i.n. with 250 colony forming units (CFU) *tdTomato* H37RV *M.tb* following establishment of human immune cell populations. Formalin fixed paraffin embedded tissue sections were cut, dewaxed and stained with antibody to human CD3. Marker expression was visualized with Fast Red substrate and images captured by brightfield microscopy. A, shown is the localization of T cells relative to a granuloma periphery and center (top left; 4X, top right; 10X). Enlarged images of T cell staining in the indicated areas are shown in the bottom panels (40X). (B) Human T cells in portal tracts and sites of inflammation in the liver (top panel, 10X) and an enlarged area showing orchestration of T cells around an inflammatory focus (bottom panel, 20X). Shown are representative images (n = 3) of animals sacrificed at 7 wk p.i.

The lymphocytic mantle, composed primarily of T lymphocytes, is a key feature associated with *M.tb* containment in the granuloma. Here, immunohistochemistry (IHC) was performed on formalin-fixed, paraffin-embedded tissue to determine if this classical feature, associated with both protective host response and immune-mediated pathology, occurs in our model. Consistent with human tissue and other animal models, T cells were characteristically organized to the outer area of sites of infection and/or inflammation. At 3 weeks p.i., human CD3^+^ cells (T cells) were detected in the periphery of lung granulomas and foci of infection in both lung and liver (Figure 6). Similarly, T-cells were also found in lesser numbers in the center of larger lung granulomas (Figure 6A) and adjacent vasculature in the lung and liver. These results suggest that the human T cells in the BLT mouse are able to respond to cytokine/chemokine gradients at sites of *M.tb* infection.

## Discussion

Understanding the human immune response to *M.tb* is critical for the development of countermeasures to prevent, diagnose, and treat TB. An animal model that would enable the study of human cell mediated immunity to *M.tb* and especially, investigations of HIV/*M.tb* co-infection would have tremendous value. Our studies show, for the first time, that the humanized BLT mouse has promise as small animal model of experimental TB. We have successfully generated BLT mice with a high level of human leukocyte reconstitution, including CD4^+^ and CD8^+^ T cells that can respond to activation with the full repertoire of antibacterial effector molecules. These mice develop productive and disseminated *M.tb* infection that causes pathology similar to that observed in human tissue and several non-rodent animal models. This represents a significant advance in our ability to study specific aspects of the human cell-mediated immune response to *M.tb*. Moreover, we expect the model to have utility in addressing the large gaps in our understanding of HIV/*M.tb* co-infection.

Due to the restricted tropism of HIV to human cells, the various humanized mouse models have been quickly adopted as the animal model of choice for HIV research. Studies key to understanding several important concepts in HIV infection have been enabled by adoption of this model, including natural routes of transmission, latency, and effectiveness of vivo antiretroviral drug prophylaxis [17], [18], [32], [33]. The use of these models has been expanded to other important infectious agents that previously were limited by the available animal models, including Dengue virus [22], [34] *Plasmodium falciparum* [35], Epstein-Barr virus [26], and Salmonella [21].

Of the various models employed for HIV research, we considered the BLT mouse developed by J. Victor Garcia-Martinez and colleagues [26] to be the most appropriate for use in developing our TB model. Previous reports indicated that human APC and functional CTL responses could be generated in response to Epstein-Barr virus infection in this model [26]. This is important because interactions between macrophages and T cells (CD4 as well as CD8) are critical in the protective host response to *M.tb*. The BLT mice we are now routinely generating in our collaborative group have a substantial reconstitution of human leukocytes, similar to previous reports in the BLT and stem cell reconstituted NSG models [20], [26], [32]. Also in agreement with previous studies, we observe circulating and tissue T cells (CD4 and CD8), monocytes/macrophages, DC, and small numbers of NK cells [18], [26].

In the BLT mouse, DC populations have been shown to constitutively express activation markers that guide the outcome of DC/T cell interactions [26]. To date, monocyte populations have only been described in the humanized mouse model where NSG mice were reconstituted with CD34^+^ stem cells in the absence of fetal thymus or liver. In that study, the CD14^+^ cells were found to be phenotypically immature and lacked expression of activation and antigen presentation markers [36]. In our studies in the BLT mouse model, CD14^+^ cells from peripheral blood of NSG BLT mice are shown to constitutively express HLA-DR, CD80, CD86, and CD40. Thus, similar to DC populations previously described [26], the monocyte/macrophage populations in the BLT mice are phenotypically mature. This is an important observation, as mononuclear phagocytes, especially macrophages, play critical roles in several stages of *M.tb* infection and host response. In support of macrophage function in humanized mouse models, a very recent study demonstrated intact phagocytic uptake and IFN-γ-mediated intracellular killing of *Salmonella typhi* [37].

Cellular immune responses by T cells are required for protective immunity to *M.tb*. The importance of secretion of IFN-γ by CD4^+^, CD8^+^, and NK cells, is firmly established through studies in gene-deleted animals and patients with rare genetic defects to be a necessary effector molecule, activating macrophages to eliminate the intracellular bacteria [38], [39]. In the BLT mouse, we observed T cell subsets (CD4, CD8) in the blood and various tissues to express low levels of constitutive as well as activation-induced IFN-γ. Similar to observations of Schultz, et al [27] in a CD34^+^ stem cell-reconstituted NSG model, we demonstrate that human CD8 T cells in the BLT mouse constitutively express perforin. Further, we were able to demonstrate that CD4, CD8 (Fig. 2), and NK cell (not shown) populations express the antimicrobial granulysin protein, as well as perforin, upon activation.

Granulysin is an important antibacterial molecule found in granules of human and cattle cytotoxic T cells and NK cells that is known to kill intracellular mycobacteria via a perforin-facilitated mechanism [40]–[43]. The lack of a granulysin gene homologue in rodents has limited studies of the role of this antibacterial molecule in protective immunity. Levels of granulysin in human plasma have been shown to correlate with successful TB recovery [44] and selective expression of granulysin in the granuloma periphery in a cattle model and human autopsy tissue indicate a potentially important role in the host response to mycobacteria in the lung [45], [46]. The potential to further study expression of granulysin during *M.tb* infection in vivo is an exciting aspect of the BLT TB model. This avenue of investigation is also important for subsequent HIV/*M.tb* co-infection studies, as activation of T and NK granulysin expression has been shown to be very susceptible to HIV-mediated suppression in patients and an in vitro model [30], [47], [48]. In subsequent studies, we will further evaluate granulysin as a potential biomarker, or part of a biosignature, that predicts the state of the host response to *M.tb*.

The use of a fluorescent isolate of *M.tb* allowed us to follow the kinetics of infection as has previously been described for traditional mouse models [31], [49]. Similar to these studies, we observed a progressive increase in bacterial burden over time in the lung though signal strength declined after 4 weeks presumably due to loss of the plasmid encoding the red fluorescent protein. Thus this technique was informative in our studies but requires further development using an isolate with a gene for fluorescent protein expression stably integrated into the *M.tb* genome. Ultimately, the optimization of this technique for use in our humanized mouse model allowed us to follow disease progression and make decisions on necropsy time points. This is especially important in this model due to the expense and limited number of animals compared to the use of traditional, non-humanized mice. In subsequent studies, the use of *in-vivo* imaging will be an important tool for visualizing the kinetics of co-infection, or experimental drug or vaccine treatments effects, using fluorescent or bioluminescent HIV and *M.tb* isolates.

The growth of *M.tb* indicated by our *in vivo* imaging in the weeks following infection was confirmed by CFU enumeration of tissue bacterial burden. The bacterial burden in the spleen and liver were generally 1 log less than in the lung at the same time point; an expected pattern of *M.tb* dissemination based on other animal models. Regardless of dose, prior to 3–4 weeks of infection, the disease pathology we observed was similar to that reported in many mouse models with small but organized foci of inflammation increasing in prominence. After 4 weeks, however, we observed a striking difference in lung pathology, with the development of large granulomatous lesions. Of note, we observed a spectrum of inflammation within individual lungs by 4 weeks of infection ranging from small foci of inflammation to large coalescing granulomas within the same lung lobe. As disease progressed in our lower dose studies these lesions expanded, coalesced, and became increasingly caseous and necrotic. The center of the growing granulomas was accellular, and AFB was primarily found in the periphery instead of the center of the lesion, consistent with human TB disease pathology in the lung.

The non-reconstituted NSG mouse did not reproduce the TB disease that we observed in the BLT mouse. These mice lacking human immune cell reconstitution developed an inflammatory disease that was characterized by small but organized foci of infection in the lung and dissemination to the spleen and liver. Surprisingly, 2 of the 4 infected animals survived to 5–6 weeks and, based on tissue pathology and bacterial load, may have survived much longer than these planned time points. By 6 weeks p.i., however, the NSG mice failed to develop the large, organized, granulomas that were observed in the BLT mice by 4 wk p.i. Though this demonstrates the contribution of the human cells for development of important features of TB pathology in the model, it is still very interesting that these severely immune-compromised animals survived several weeks post infection. Traditional mouse models with targeted deletions of important immune mechanisms, such as the IFN-γ deficient (GKO) mice that are highly susceptible to *M.tb,* succumb to the infection within 5–8 weeks after a low dose inoculum of 50–200 CFU [39], [50]. The Kramnik (C3HeB/FeJ) mouse model, which is also highly susceptible to *M.tb*, succumb to the disease within 4–12 weeks depending on dose [10], [51]. Like these models, the NSG mouse retains functional murine neutrophils which have an increasingly appreciated role in controlling infection with intracellular bacteria.

A critical observation in our studies was the granuloma lesion with central necrosis; considered the hallmark of human tuberculosis infection [10], [52], [50]. Non-human primates and guinea pigs do form the classic granulomatous lung response and are thus considered excellent models to study TB pathogenesis. Widely used mouse models of *M.tb* infection, such as the BALB/c and C57BL/6, do not show necrotic granuloma lesions or a spectrum of lesions that is associated with human disease [52], [10], [50]. Necrotic granuloma lesions have been observed in immunosuppressed mouse models including the GKO and C3HeB/FeJ model [10], [39], [51], [52] or mice treated with Trehalose 6,6′-dimycolate [53], an *M.tb* cell wall glycolipid that initiates a CD3^+^T cell-mediated inflammatory response [54].

Another interesting feature of our model is the cholesterol crystal formation in the lung lesions that we observed. Cholesterol catabolism is important for *M.tb* growth and genes specific to cholesterol and general lipid metabolism have been identified as part of the differential *M.tb* transcriptome specific to intracellular growth in macrophages [55]. We additionally observed frequent bronchial obstructions that were dense in AFB, a characteristic feature of human lung TB that, to our knowledge, has not been previously observed in mouse models. Bronchiole obstruction in afflicted humans is believed to be important in disease transmission due to expulsion of AFB upon coughing [11], [56]. Endobronchial *M.tb* is thought to be an indicator of bacterial spread in the lungs and an early indicator of post-primary TB [57]. Though mice lack the cough reflex, the development of the obstruction is an important endpoint in the model and will allow investigation of this hallmark of human disease in the animal model. In contrast to the GP and NHP models, we did not observe fibrosis or calcification in any animals infected with *M.tb*. However, in these initial studies we did not allow infection to progress beyond 6 weeks in most experiments, with the maximum time point of 8 weeks. Future studies with an extended time frame and aerosol-delivery of infection will be performed to study survival and pathology at later stages of infection.

Importantly, in our studies, human T cells were found at the granuloma periphery; a controversial feature of disease with regards to bacterial containment, on-going antigen presentation, and immune-mediated pathology [58]. The recruitment of T cells to these sites of infection will enables several avenues of investigation important for vaccine design, including studies of human memory T cell populations and effector molecule profiles associated with specific pathology. It will additionally allow us to evaluate the impact of HIV infection on T cell mediated immunity in the lungs. Upon further optimization of infection, it may additionally be possible to develop a latency model, similar to the Cornell model [59], in the BLT mouse.

In summary, our studies support further exploration of the humanized BLT mouse for basic and translational TB research. The expense of generating BLT mice is a limitation compared to traditional mouse, guinea pig, and rabbit models; although, compared to the expense of the NHP, BLT mice are considerably cheaper. Additionally, the availability of human reagents including antibodies, multi-plex protein arrays, and HLA-matched tetramers/pentamers is a significant advantage to the model. An important application of this model specific to our laboratory’s interests will be the further development of an HIV/*M.tb* co-infection model. Further development of this co-infection model could generate a powerful research tool for use to understand the pathobiology of co-infection and to test medical countermeasures.

## Materials and Methods

### Ethics Statement

All animal procedures were performed in accordance with the regulations of the NIH Office of Laboratory Animal Welfare and were approved by the University of Texas Medical Branch (UTMB) Institutional Animal Care and Use Committee (IACUC). Discarded tissue from deceased human fetuses was obtained via a non-profit partner (Advanced Bioscience Resources, Alameda, CA) as approved under exemption 4 in the HHS regulations (45 CFR Part 46).

### Generation of Humanized BLT Mice

NOD.Cg-*Prkdc^scid^ Il2rg^tm1Wjl^*/SzJ mice, also known as NOD/SCID/γ_c_^null^ or NSG mice (Jackson Laboratories), 3–5 weeks of age, were housed in a specific pathogen-free microisolator environment. Mice were engrafted with human fetal liver and thymus (Advanced Bioscience Resources, Alameda, CA) tissue after receiving 200 cGy of irradiation at a rate of 119 cGy/min (RS-200 Rad Source, Suwanee, GA) as previously described [26]. Following engraftment of tissue, mice were intravenously (i.v) injected with approximately 1×10^6^ hematopoietic stem cells (CD34^+^ cells) per mouse from the same human fetal tissue donor. For two weeks after implantation, mice received acidified drinking water (pH 3.0) with antibiotics. Twelve weeks post-engraftment, human leukocyte reconstitution was evaluated in peripheral blood.

### Animal Infections

*M.tb (tdTomato* H37Rv) was propagated by growth to log phase in Middlebrook 7H9 broth (Becton, Dickinson, and Company, Sparks, MD, USA) supplemented with 50% glycerol (Sigma, St. Louis, MO, USA), BBL Middlebrook albumin dextrose complex enrichment (ADC, BD), and 20% Tween 80 (Fisher Scientific, Fair Lawn, NJ, USA) as previously described [60]. Mice were infected intranasally with 40 µl (20 µl/nare) of logarithmically decreasing doses (10^6^–10^2^ CFU) of *M.tb tdTomato* H37Rv diluted in Dulbecco’s Phosphate-Buffered Saline (PBS, Cellgro, Manassas, VA, USA). The actual dose was further confirmed by CFU enumeration of inoculum suspension. At specified time points, mice were euthanasized using isoflurane (Primal Critical Care, Inc., Bethlehem, PA) overdose. Cervical dislocation was subsequently performed to ensure death, as approved by the UTMB-IACUC. All animal experiments and work with *M.tb* were performed in a CDC-approved animal biological safety level-3 (ABSL-3) and BSL3 facilities in the Galveston National Laboratory in accordance with biosafety procedures approved by the UTMB Environmental Health and Safety Division.

### In-vivo Imaging

Mice were anesthetized with 3% isoflurane (Piramal Critical Care, Inc. Bethlehem, PA, USA) in an oxygen-filled induction chamber, transferred to an isolation chamber, and placed in the imaging chamber which contains an integrated anesthesia system. Anesthesia was continued to be administered at 1–2% isoflurane. *In-vivo* fluorescent images were acquired using the In-Vivo Imaging System (IVIS) Spectrum (Caliper Corporation, Alameda, CA, USA). Images were acquired at each excitation and wavelength pair as previously described [61]. The fluorescent signal was obtained at a 535 nm and 605 nm excitation light and read at 580, 620, 640, 660, 680, 700 nm. Compensation was applied to correct for autofluorescence.

### Isolation of PBMCs and Leukocyte Analysis

Reconstitution levels of lymphocytes and monocytes were determined pre- and post-infection by multi-variate flow cytometry. Peripheral blood was collected from the tail vein of humanized BLT mice and placed in 500 µl 3 mM ethylenediaminetetraacetic acid (EDTA, Capitol Scientific, Austin, TX, USA). Peripheral blood mononuclear cells (PBMCs) were isolated by incubation with Red Blood Cell Lysis Buffer (Sigma, St. Louis, MO, USA) as recommended by manufacturer. Subsequently, PBMC were incubated with CD16/CD32 Fc Block (BD Biosciences, San Jose, CA, USA) to reduce non-specific binding of antibodies. Cells were labeled with directly conjugated antibodies specific to human lymphocyte surface markers: PE-Cy7 CD45, Alexa Fluor 700 CD56, APC Cy7 CD3, Pacific Blue CD4, and PerCP-Cy5.5 CD8 (BD Biosciences) and is a separate experiment, γδTCR (eBioscience). In some experiments PBMC were labeled with antibody to human antigen presenting cell phenotype and activation markers: APC-Cy7 CD14, PE-Cy7 CD40, PerCP-Cy5.5 HLA-DR, FITC CD80, and PE CD86 γδTCR (BD Bioscience). For determination of effector function, spleens from non-infected humanized mice were obtained and processed to single-cell suspensions by pressing through a 70 µ filter. Following incubation with Red Blood Cell Lysis Buffer, PBMCs were maintained in complete culture medium (cRPMI), RPMI 1640 supplemented with 10% fetal bovine serum (FSB), 2 mM L-glutamine, 1 mM sodium pyruvate, 0.1 mM nonessential amino acids, and 1% penicillin-streptomycin (Life Technologies, Grand Island, NY, USA) and stimulated with IL-15 (15 ng/ml) or CD3/28, a T-cell stimulant, for 5 days (as recommended by the manufacturer Life Technologies),. Cells were treated with GolgiStop (BD Bioscience) during the last 5 hours of culture, washed, and subsequently labeled using antibodies to human lymphocyte surface markers. Cells were permeabilized using the BD Cytofix/Cytoperm kit (BD Bioscience), then labeled with antibodies specific to human APC IFN-γ, FITC granulysin, PE-Perforin, or PE Ki-67, a proliferation marker (BD Bioscience). Samples were finally incubated for 48 hours in 2% formaldehyde (Polysciences Inc, Warrington, PA, USA) diluted in PBS prior to acquisition. A total of 50,000 gated events (based on expected leukocyte side scatter/forward scatter characteristics) were collected using a BD LSR II (Fortessa) flow cytometer (BD Biosciences). Analysis of data was performed by FCS Express (De Novo, Los Angeles, CA, USA) software. To control for background and to establish thresholds for gating positive cells, isotype matched antibodies labeled with the same fluorochromes were used. Cells were selected based on the human CD45 marker and further analyzed for human CD3, CD4, CD8, CD56, and CD14.

### CFU Enumeration

At specified time points, following aseptic removal of organs, half of the liver, lungs, and spleen were placed into 1 ml of PBS in 50 ml small tissue grinders (Kendall, Mansfield, MA, USA). Following homogenization of tissues, serial dilutions of organ samples were prepared in PBS, and 6–5 µl droplets were placed on 7H11 agar plates (BD Biosciences) as previously described [42]. All studies were performed in a CDC-approved biological safety level-3 (BSL-3) facility.

### Pathology Assessment

At specified time points, following aseptic removal of organs, the remaining half of tissues were placed in 10% Neutral Buffered Formalin (Statlab, McKinney, TX, USA) for 48 hours to inactivate infectious agent, changed after 24 hours, and finally stored in 70% ethanol. Tissues were embedded in paraffin then stained with hematoxylin and eosin (H&E) and additional sections were stained using the Ziehl-Neelson method to visualize acid-fact bacteria (AFB) to detect *M.tb* bacilli. Processing and staining were performed at the University of Texas Medical Branch Research Histopathology Core. Paraffin embedded tissue samples were further stained using EnVision™ G|2 System/AP, Rabbit/Mouse (Permanent Red) (Dako, Carpinteria, CA) for human CD3 (Dako) according to manufacture recommendations. Lung, liver, and spleen tissue was evaluated by a trained pathologist with expertise in tuberculosis disease progression, with confirmation done in a slide blinded manner by pathologists at the UT-Houston Medical School.

## Supporting Information

Figure S1 **Tissue distribution of leukocytes in humanized BLT mice.** Tissues (lung, spleen and liver) from non-infected animals were disrupted to single-cell suspensions and analyzed by flow cytometry. Total human leukocytes were gated based on CD45 expression and further analyzed for phenotype using antibodies specific to human CD4, CD8, CD56, and CD14. Shown is the percentage of the total cells that express human CD45, and the % of the gated cells that express markers that identify T cell subsets (CD4, CD8), NK cells (CD56), and monocyte/macrophages (CD14). Data are from a representative BLT mouse within the groups described in Figure 1.
(TIF)

Figure S2 ***M.tb* infection in non-reconstituted NSG mice differs from that observed in humanized mice.** NSG mice that were not engrafted with human tissue or stem cells were infected i.n. with *M.tb*. Shown are images captured by brightfield microscopy following staining of infected tissues using H&E and acid fast stain**.** Shown in A are lung, and B, liver, tissue pathology and localization of AFB at 2 and 6 weeks p.i. (H&E 4X, AFB 40X). Results are representative of mice sacrificed at 2 and 6 wk p.i.
(TIF)
