# Supplementary figures and images for: A Humanized Mouse Model of Tuberculosis
Source: PLoS One. 2013 May 17;8(5):e63331. doi: 10.1371/journal.pone.0063331 (PMC3656943; doi:10.1371/journal.pone.0063331)

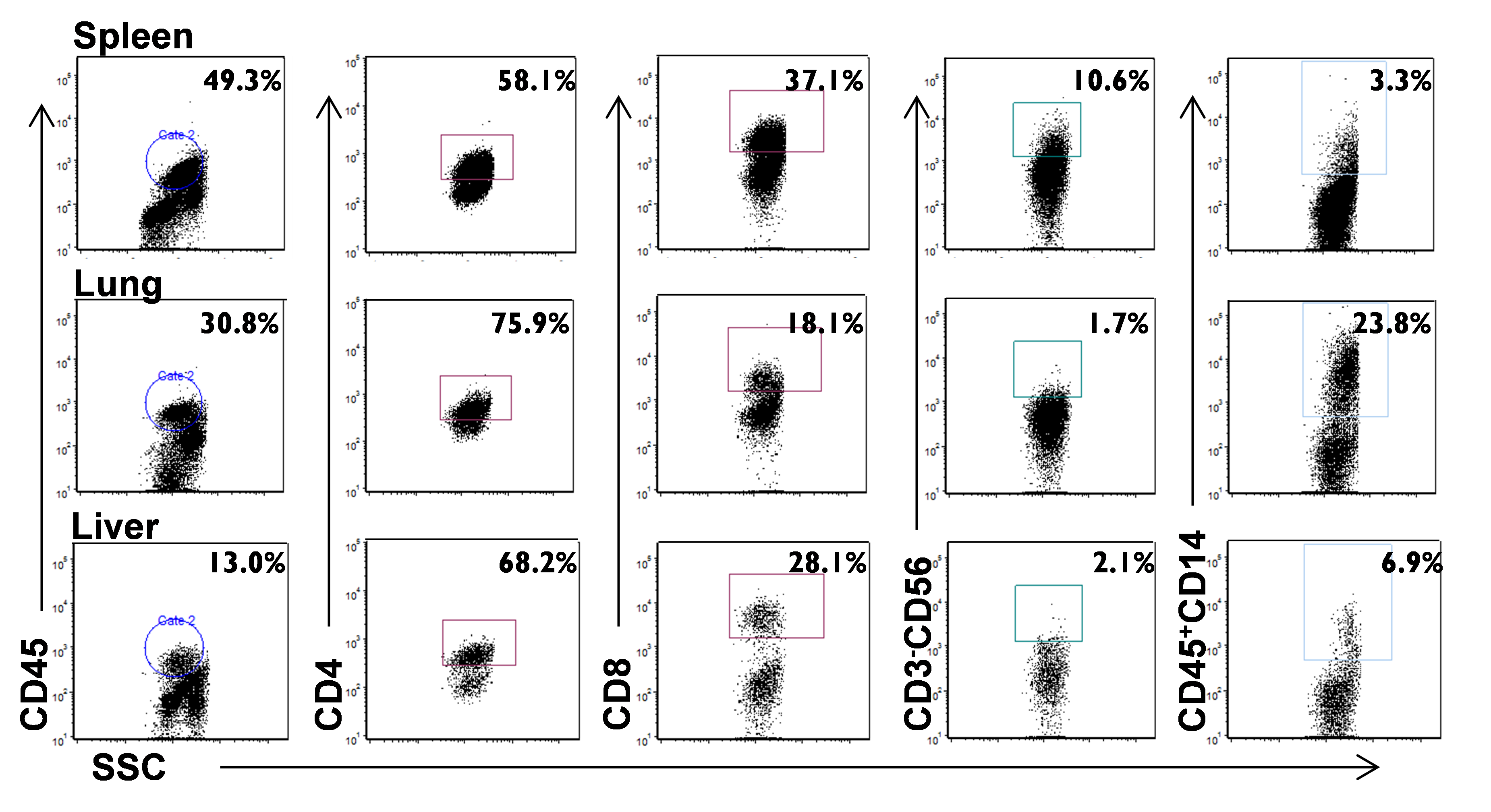

Supplement: Figure S1 — Tissue distribution of leukocytes in humanized BLT mice. Tissues (lung, spleen and liver) from non-infected animals were disrupted to single-cell suspensions and analyzed by flow cytometry. Total human leukocytes were gated based on CD45 expression and further analyzed for phenotype using antibodies specific to human CD4, CD8, CD56, and CD14. Shown is the percentage of the total cells that express human CD45, and the % of the gated cells that express markers that identify T cell subsets (CD4, CD8), NK cells (CD56), and monocyte/macrophages (CD14). Data are from a representative BLT mouse within the groups described in Figure 1. (TIF) [file pone.0063331.s001.tif]

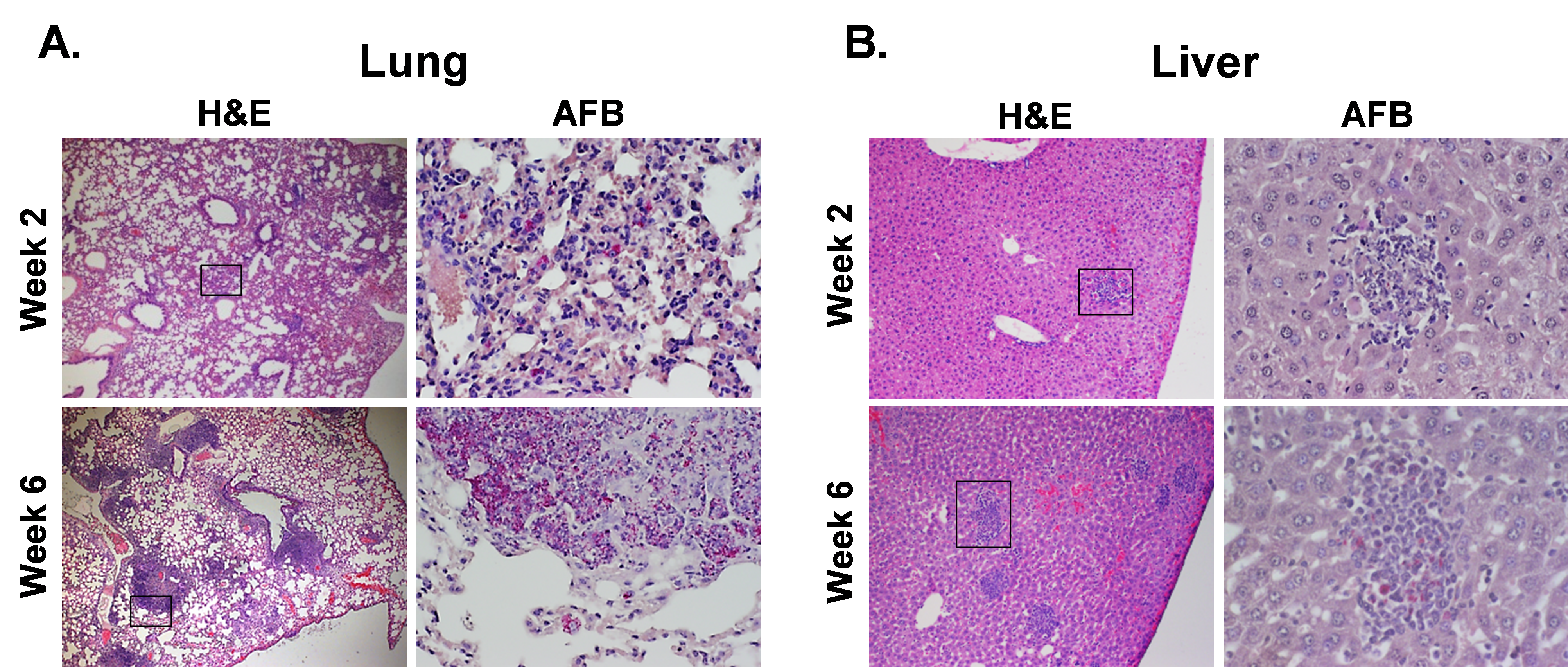

Supplement: Figure S2 — M.tb infection in non-reconstituted NSG mice differs from that observed in humanized mice. NSG mice that were not engrafted with human tissue or stem cells were infected i.n. with M.tb. Shown are images captured by brightfield microscopy following staining of infected tissues using H&E and acid fast stain. Shown in A are lung, and B, liver, tissue pathology and localization of AFB at 2 and 6 weeks p.i. (H&E 4X, AFB 40X). Results are representative of mice sacrificed at 2 and 6 wk p.i. (TIF) [file pone.0063331.s002.tif]
